# Genetic Differentiation between Natural and Hatchery Stocks of Japanese Scallop *(Mizuhopecten yessoensis*) as Revealed by AFLP Analysis

**DOI:** 10.3390/ijms11103933

**Published:** 2010-10-15

**Authors:** Wei-Dong Liu, Hong-Jun Li, Xiang-Bo Bao, Xiang-gang Gao, Yun-feng Li, Chong-Bo He, Zhan-Jiang Liu

**Affiliations:** 1 Liaoning Ocean and Fisheries Science Research Institute, The Key Laboratory of Marine Fishery Molecular Biology of Liaoning province, Dalian 116023, China; 2 Institute of Chemical and Physics, Chinese Academy of Sciences, Dalian 116023, China; 3 College of Agriculture, Auburn University, Auburn, AL 36849, USA

**Keywords:** Japanese scallop, Mizuhopecten yessoensis, AFLP, genetic diversity, population structure, selective breeding

## Abstract

Japanese scallop (*Mizuhopecten yessoensis*) is a cold-tolerant bivalve that was introduced to China for aquaculture in 1982. In this study, amplified fragment length polymorphism (AFLP) markers were used to investigate levels of genetic diversity within *M. yessoensis* cultured stocks and compare them with wild populations. Six pairs of primer combinations generated 368 loci among 332 individuals, in four cultured and three wild populations. High polymorphism at AFLP markers was found within both cultured and wild *M. yessoensis* populations. The percentage of polymorphic loci ranged from 61.04% to 72.08%, while the mean heterozygosity ranged from 0.2116 to 0.2596. Compared with wild populations, the four hatchery populations showed significant genetic changes, such as lower expected heterozygosity and percentage of polymorphic loci, and smaller frequency of private alleles, all indicative of a reduction in genetic diversity. Some genetic structures were associated with the geographical distribution of samples; with all samples from Dalian and Japan being closely related, while the population from Russia fell into a distinct clade in the phylogenetic analysis. The genetic information derived from this study indicated that intentional or accidental release of selected Japanese scallops into natural sea areas might result in disturbance of local gene pools and loss of genetic variability. We recommend monitoring the genetic variability of selected hatchery populations to enhance conservation of natural Japanese scallop resources.

## 1. Introduction

Amplified fragment length polymorphism (AFLP) is a multilocus marker technique developed by Vos *et al.* [1], which combines the strengths of restriction fragment length polymorphism (RFLP) and random amplification of polymorphic DNA (RAPD). It does not require any previously known genetic information, a feature especially useful for species with limited sequence resources [2,3]. Moreover, AFLP has the capability to produce multi-locus fingerprints in a single polymerase chain reaction (PCR) analysis, significantly reducing the cost of analysis and increasing the possibility of detecting polymorphisms. Because of such advantages, AFLP markers have emerged as a major type of genetic marker with wide applications in genetic diversity, population structure and construction of linkage maps [4].

The Japanese scallop (*Mizuhopecten yessoensis*) has a wide distribution on the cold coasts of northern Japanese islands, in the northern part of the Korean Peninsula, Russian Primorye, Sakhalin, and Kuril Islands, [5]. In 1982, *M. yessoensis* (332 individuals) were first introduced into China from Japan by Liaoning Ocean and Fisheries Science Research Institute [6] as an alternative species for aquaculture in northern China. Japanese scallop quickly became a popular species because it is larger in size and commands a higher market value than the native zhikong scallop (*Chlamys farreri*) and the introduced bay scallop (*Argopecten irradians*). By 2005, the annual production of Japanese scallop had reached about 150,000 tons in China [7]. Chinese *M. yessoensis* farming is mainly distributed in the region of Liaoning and Shandong provinces, and Dalian (Liaoning) scallop aquaculture accounts for about 90% of the total national production [8].

The Japanese scallop industry now relies mainly on hatchery produced spat in China. Each year, hatcheries use cultured stocks as the brood stock to produce the next generation for culture, which has been the practice for nearly three decades. Another seed production method is natural spat collection. Scallop farmers use settlement substratum to collect larvae in spring, and can attain a lower mortality rate using natural spat than hatchery produced seeds [8]. In this regard, there are several concerns for the sustainability of the scallop aquaculture, including inbreeding among the individuals derived from artificial seeds and genetic impact of stocked hatchery scallops on the natural populations. Several recent studies have begun to address these questions including genetic analysis of Japanese scallops using isozyme markers [9], mitochondrial DNA [10,11] and a limited number of microsatellite markers [7,12]. In this study, we conducted genetic diversity analysis using AFLP markers to provide a greater coverage of genomic scope for the assessment of variations in wild and farmed populations of *M. yessoensis*.

## 2. Materials and Methods

### 2.1. Scallop Samples

**Figure 1:**
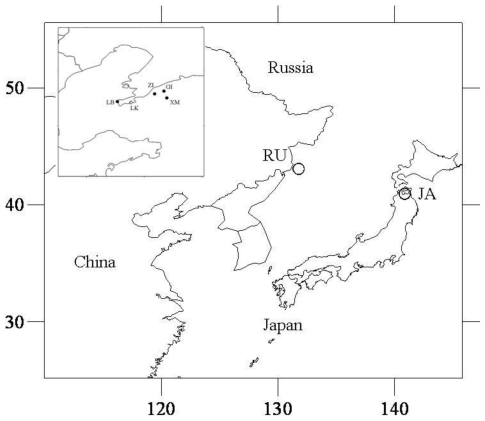
Map showing locations and abbreviated names for four hatchery samples (●) and three natural (○) Japanese scallop samples.

We surveyed three natural and four cultured populations of the Japanese scallop *M. yessoensis* (Figure 1; Table 1). The natural Japanese scallop populations were derived from Aomori (JA, Japan), Vladivostok (RU, Russia), and Lüshunkou (LSK, China). Random samples of cultured 2 year old scallops were collected from commercial scallop farms in four localities around Dalian in Liaoning Province: Guanglu Island (GI), Lingshui Bridge (LB), Zhangzi Island (ZI) and Xiaochang Mountain (XM). GI, LB and ZI populations were founded in the early 1980s using introduced stocks from JA, and no selected strains or lines have been established. The LSK population experienced massive summer mortality and only 10% individuals survived. The XM population is composed of second generation offspring derived from hybridization between the ZI stocks and the wild population introduced from JA in 1998. A muscle sample from each scallop was preserved in 80% ethanol for genomic DNA isolation.

### 2.2. DNA Isolation and AFLP Analysis

Genomic DNA was extracted from muscle samples with the traditional phenol-chloroform method [13]. DNA concentrations were estimated and standardized against a known concentration of λ DNA marker on 1.0% agarose gel, and adjusted to 100 ng/μL. AFLP analysis was performed essentially as described by Vos *et al.* (1995) [1]. Genomic DNA (about 500 ng) was digested with restriction enzymes EcoRI and MseI before ligation with restriction site-specific adaptors. Pre-selective amplification was carried out using adaptor specific primers without a selective base. The pre-selective PCR products were diluted 30-fold and used as template DNA in selective amplifications. Selective amplification was performed using selective primers with three selective nucleotides at the 3′ end. Six primer combinations were used (E = *Eco*RI, M = *Mse* I): E-AAC/M-CCA, E-ACA/M-CTC, E-ATC/M-CCT, E-ATC/M-CTT, E-ATG/M-CCT, and E-ATG/M-CTG (Table 2). The PCR products were separated on 6% denaturing polyacrylamide gel with 7.5 M urea at 60 W constant power for 2.5 h, according to the standard method described for DNA sequencing [14]. AFLP patterns were visualized by silver staining. A 100 bp DNA ladder (Takara) was used as a reference marker for allele size determination. The silver stained AFLP bands in each gel were scored as present (1) or absent (0) and only distinct polymorphic bands were scored. AFLP fragments with similar electrophoretic mobilities were assumed to be allelic and those with different mobilities were assumed to be non-allelic.

### 2.3. Data Analysis

Fragment data were transferred to a binary (1/0) data matrix. Average heterozygosities (*H*), percent polymorphic loci (*P*), and *F*_ST_ values were estimated using the TFPGA program [15]. The confidence of branch support was then evaluated by way of bootstrap analysis with 1000 replications, performed with the PAUP software package [16]. Average heterozygosity estimates were calculated for each locus and then averaged overall loci according to Nei’s unbiased heterozygosity [17]. Confidence intervals were generated by bootstrapping analysis at the 99% confidence level with 1000 replications. Genetic distances between populations were calculated by Nei’s unbiased distance and identity measures. The similarity matrix produced by TFPGA was then imported into the NTSYSpc software [18] to produce a similarity tree showing the relationships between samples.

## 3. Results

### 3.1. AFLP Polymorphism and Genetic Variation

Six AFLP primer combinations were used to genotype 332 scallops from 7 populations. Of the 350 original individuals, 18 scallops were omitted from the analysis because of a high incidence of questionable bands, presumably because of low quality DNA. A total of 368 bands were identified across the remaining 332 scallops, 337 of which were polymorphic bands, with an overall mean heterozygosity of 0.2601 (Table 3). Some variations were observed among populations with regard to genetic diversity as indicated by the average heterozygosity and percentage of polymorphic loci. The population with the greatest percent polymorphism (72%) and highest average heterozygosity (0.2596) was the natural seed population JA from Japan. The population with the lowest percent polymorphism (61%) and the lowest average heterozygosity (0.2116) was the natural seed population RU from Russia. The four artificial seed populations from Dalian were found to contain percent polymorphic loci (62–67 *vs.* 66%) and mean heterozygosity values (0.2285–0.2442 *vs.* 0.2380) similar to the natural seed population LSK from Dalian. But natural seed population JA was observed to have significantly higher percent polymorphic loci (72% *vs.* 62–67%, *P* < 0.05) and mean heterozygosity values (0.2596 *vs.* 0.2285–0.2442, *P* < 0.05) than all the populations from Dalian, indicating that higher genetic diversity existed in JA population than in those in Dalian. This may be due to a very large natural scallop population existing in Japan, while all populations around Dalian originated from a limited number of stocks introduced in the 1980s from Japan [8,19].

### 3.2. Population Structure

**Figure 2:**
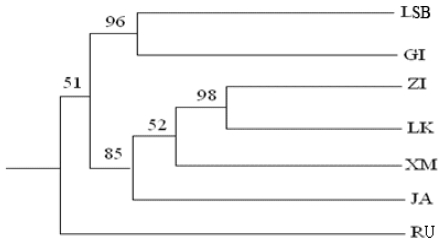
Population-wise similarity tree revealed by UPGMA cluster analysis using the Nei (1978) unbiased genetic identity measure. Significant bootstrapping values are indicated on clades.

One of the objectives of this research was to provide an initial determination of the genetic structure of the scallop population. The genetic similarity analysis, using Nei’s genetic distance with UPGMA method, revealed significant differentiations among the scallop populations (Figure 2). All populations except RU, regardless of their seed breeding methods, were clustered as a single clade, indicating their close relationship, which was congruent with results above. However, the relationship between some populations expressed difference. For instance, populations ZI and LSK were the most closely related populations, with a bootstrapping confidence value of 98%. Populations LB and GI also had a close relationship with high bootstrapping values. Population XM was comprised of hybrid offspring between paternal individuals of a newly introduced population from Aomori, Japan (JA) and maternal bottom-cultured individuals from ZI. Therefore, the closest relationships were found between population JA and ZI or LSK.

## 4. Discussion

This research uses AFLP markers for the first time for the analysis of genetic resources of Japanese scallop. Because of the large numbers of bands and high levels of polymorphism, AFLP analysis allows large genome coverage with limited resources [4]. Before this study, some initial analyses were conducted for genetic resource assessment using allozyme markers among the cultured populations [9], and microsatellite markers for the analysis of genetic resources between cultured and natural populations of Japanese scallop [20]. However, previous research has not addressed the issue of genetic diversity between artificial and natural seed populations of this species. In this study, we found lower genetic diversity in populations from Dalian than Japan, as indicated by the values of average heterozygosities and percentages of polymorphic loci. The reduced genetic variation may be attributable to some factors such as geographic isolation and genetic drift [21], and limited effective population size [22], but the major reason was the founder effect, as a limited number of individuals were imported for broodstock for the first time. Only 332 individuals of Japanese scallop were used as broodstock when introduced to Dalian, China during the years 1982–1983 [6,19], and the juvenile or “spat” scallops reproduced by these scallops were then distributed to different hatcheries for use as broodstock in the subsequent artificial breeding processes. Therefore, all the scallop populations (except for a few scallops introduced by individual hatcheries in 1998) cultured in Dalian today are the progeny of the small and limited population introduced in the past, which may be the main reason for the reduced genetic diversity of Chinese Japanese scallop culture stocks. The limited effective population size employed in artificial breeding operations has played an important role in the reduction of genetic diversity in the populations of flat oyster *Ostrea edulis* [23] and Pacific abalone *Haliotis discus* [24], but our results showed that five populations (four artificial seed populations and one natural seed population) contained similar percent polymorphic loci (62–67%) and mean heterozygosity values (0.2285–0.2442), indicating no significant variations between artificial and natural seed populations. Such similarities apparently resulted from the advanced and unique artificial seed breeding methods used, in which large numbers of parental scallops (usually 4000–8000 individuals) were employed by hatcheries to minimize the genetic effect of “bottleneck” or “genetic-drift”.

The results of the UPGMA dendrogram analyses indicated that there was population differentiation in the scallop populations studied. Population RU was separated from other populations and formed a single clade. All other populations, regardless of their seed breeding methods, were clustered into the other single clade, indicating their geographic differentiation. One interesting finding was that the natural seed population LSK clustered closely together with the artificial population ZI with a high bootstrapping confidence value, despite the geographic distance (about 100 km) between the two farming locations. It is possible that the natural seed population LSK was spawned or reproduced by the scallop population in Zhangzi Island and then drifted with the ocean currents to the Lüshun Kou area. Because no Japanese scallop population previously inhabited the Lüshun Kou coast, the natural spats or juveniles collected must come from somewhere else nearby. Our recent driftage test (unpublished data) has shown that there is an ocean current flowing from Zhangzi Island to the Lüshunkou area that lasts about 20 days in spring (from February to March). Further research is needed to test this hypothesis. Population XM is comprised of the hybrid scallops produced by female scallops of the population originally introduced in the 1980s crossed with male scallops imported in 1998 from Aomori, Japan. Population LSB was also made up of scallops cultured by suspended cage culture, and it clustered in the same clade. The population LSB and GI clustered together, and there may have been broodstock exchanges between the hatcheries in LSB and GI in the past.

## 5. Conclusions

In the present study, we used AFLP markers to investigate levels of genetic diversity within four *M. yessoensis* cultured stocks and compared them with three wild populations. The results indicate that some differentiation existed between wild and cultured populations, even though the ancestors of the currently cultured scallops were introduced from Aomory, Japan, and artificial breeding operations have had no substantial effect on the genetic structure of the scallop populations examined in Dalian. The data can be used as baseline information for further study of the interactions between hatchery based artificial breeding and natural seed collection of Japanese scallop in China.

## Figures and Tables

**Table 1:** Samples used in the present study.

No.	Abbreviation	Location	Nature of seed	Culture method
1	GI	Guanglu Island	artificial	suspended
2	ZI	Zhangzi Island	artificial	bottom
3	XM	Xiaochang Mountain	artificial	suspended
4	LSB	Lingshui Bridge	artificial	suspended
5	LSK	Lüshun Kou	natural	suspended
6	JA	Aomori, Japan	natural	suspended
7	RU	Vladivostok, Russia	natural	bottom

**Table 2:** Adaptors and six primer combination sequences used in the study.

Adaptors & Primers	Sequences
Adaptors	
*MesI* adaptor	GACGATGAGTCCTGAG/TACTCAGGACTCAT
*EcoRI* adaptor	CTCGTAGACTGCGTACC/CTGACGCATGGTTAA
Primers	
E-AAC/M-CCA	GACTGCGTACCAATTCAAC/GATGAGTCCTGAGTAACCA
E-ACA/M-CTC	GACTGCGTACCAATTCACA/GATGAGTCCTGAGTAACTC
E-ATC/M-CCT	GACTGCGTACCAATTCATC/GATGAGTCCTGAGTAACCT
E-ATC/M-CTT	GACTGCGTACCAATTCATC/GATGAGTCCTGAGTAACTT
E-ATG/M-CCT	GACTGCGTACCAATTCATG/GATGAGTCCTGAGTAACCT
E-ATG/M-CTG	GACTGCGTACCAATTCATG/GATGAGTCCTGAGTAACTG

**Table 3:** Average heterozygosity and percentage polymorphism for Japanese scallop across 368 AFLP loci.

Population ID	Number of sample (N)	Average heterozygosity (H)	Percentage of polymorphic loci (P)
GI	48	0.2285	64.29%
ZI	47	0.2317	62.01%
XM	48	0.2300	62.66%
LSB	47	0.2442	67.21%
LSK	49	0.2380	65.58%
JA	49	0.2596	72.08%
RU	44	0.2116	61.04%
Overall	332	0.2601	91.58%
